# The indispensable human factor in EEG-based artificial intelligence

**DOI:** 10.1002/epi.70085

**Published:** 2026-01-24

**Authors:** Fábio A. Nascimento, M. Brandon Westover

**Affiliations:** 1 Department of Neurology, Washington University School of Medicine, St. Louis, Missouri, USA; 2 Department of Neurology, Beth Israel Deaconess Medical Center, Boston, Massachusetts, USA

Artificial intelligence (AI) is rapidly transforming many fields. In electroencephalography (EEG) and epilepsy, recent advances hold significant promise for clinical care and research. Several AI models can now interpret select aspects of diagnostic EEG recordings with performance comparable to, and at times surpassing, that of human experts.^1,2^ Potential benefits include helping to (i) address geographic shortages of EEG expertise, (ii) manage growing volumes of EEG recordings in clinical practice, and (iii) enhance EEG/epilepsy education.^3^ Yet this technology remains fundamentally dependent on human intelligence for data annotation, model validation, and clinical oversight.

The performance of AI models is tightly linked to the quality of their training data, which is virtually always annotated by human readers. Inaccurate or inconsistent labeling degrades model performance - the classic “garbage in, garbage out” problem. Therefore, standardized terminology and uniform interpretative approaches are essential. In other AI domains, such as large language models, the detrimental effects of labeling noise can be mitigated by training on billions of examples. EEG AI models, by contrast, are typically trained on tens of thousands of studies or fewer, far below the threshold where scale can compensate for variability. This is partly because most of the world’s EEG data remains locked away in hospital archives and unavailable for model development.

AI development is further constrained by the paucity of robust external reference standards. Although long-term video-EEG recordings and long-term outcome data provide high-quality labels, they are available only for select patient subsets. Consequently, expert consensus often becomes the *de facto* gold standard.^4^ This further underscores the importance of skilled human readers, whose expertise forms the foundation for accurate model training and validation. Moreover, the construction of AI systems requires judicious human supervision to ensure that training data are representative and unbiased, validation methods are rigorous and can detect failures, and interpretability is prioritized so that AI architectures can be understood and properly audited by the medical community.

Human oversight is especially critical when AI systems encounter “corner cases.” These include actionable but atypical abnormalities - such as seizures or epileptiform discharges with unusual morphology, uncommon evolution, and/or prominent concomitant artifact - as well as artifacts that mimic ictal or interictal epileptiform patterns. Such cases require reasoning from first principles, knowledge of neurophysiology, and extensive experience. Because they are rare, they are sparsely represented in existing training datasets, leaving current AI tools ill-equipped to identify them reliably.

The overall pattern space in EEG is also far broader than what current models address. A recent effort identified 68 essential EEG findings^5^; yet current AI tools handle no more than a handful. The 2021 American Clinical Neurophysiology Society’s Standardized Critical Care EEG Terminology^6^ prescribes more than 100 distinct pattern combinations, most with no large, well-labeled datasets available.

Human electroencephalographers, therefore, remain indispensable - for data annotation, model validation, and clinical oversight. They are equally vital for training the next generations of readers and preserving EEG expertise as AI models increasingly automate clinical practice. In addition, the human workforce is integral for guiding responsible AI development and implementation - including addressing technical nuances such as ensuring equitable access to AI infrastructure across different geographies. At the same time, global demand for reliable EEG interpretation will never be met by human specialists alone. As a result, human–AI partnerships will be necessary to bridge this gap and meet the increasing clinical needs.

We advocate that sustained investment in both human expertise and large-scale data infrastructure is paramount for developing AI systems that can be safely, effectively, and equitably deployed in clinical practice. From a human standpoint, efforts should focus on ensuring that electroencephalographers are well trained and as “noise-free”^7^ as possible. From a data structure standpoint, efforts should prioritize the curation of large, diverse, and shareable EEG datasets that capture the full spectrum of patterns, including rare but critical presentations. This achievement will require coordinated international collaboration to unlock archived data, harmonize data formats and annotations, and contribute to shared repositories.

## Data Availability

Data sharing is not applicable to this article as no datasets were generated or analyzed.
